# ATP11C Facilitates Phospholipid Translocation across the Plasma Membrane of All Leukocytes

**DOI:** 10.1371/journal.pone.0146774

**Published:** 2016-01-22

**Authors:** Mehmet Yabas, Weidong Jing, Sarah Shafik, Stefan Bröer, Anselm Enders

**Affiliations:** 1 Department of Immunology and Infectious Disease, The John Curtin School of Medical Research, The Australian National University, Canberra, ACT, Australia; 2 Department of Genetics and Bioengineering, Faculty of Engineering, Trakya University, Edirne, Turkey; 3 Division of Biomedical Science and Biochemistry, Research School of Biology, The Australian National University, Canberra, ACT, Australia; Cornell University, UNITED STATES

## Abstract

Organization of the plasma membrane into specialized substructures in different blood lineages facilitates important biological functions including proper localization of receptors at the plasma membrane as well as the initiation of crucial intracellular signaling cascades. The eukaryotic plasma membrane is a lipid bilayer that consists of asymmetrically distributed phospholipids. This asymmetry is actively maintained by membrane-embedded lipid transporters, but there is only limited data available about the molecular identity of the predominantly active transporters and their substrate specificity in different leukocyte subsets. We demonstrate here that the P4-type ATPase ATP11C mediates significant flippase activity in all murine leukocyte subsets. Loss of ATP11C resulted in a defective internalization of phosphatidylserine (PS) and phosphatidylethanolamine (PE) in comparison to control cells. The diminished flippase activity caused increased PS exposure on 7-aminoactinomycin D^−^ (7-AAD^−^) viable pro-B cells freshly isolated from the bone marrow of ATP11C-deficient mice, which was corrected upon a 2-hour resting period *in vitro*. Despite the impaired flippase activity in all immune cell subsets, the only other blood cell type with an accumulation of PS on the surface were viable 7-AAD^−^ developing T cells but this did not result in any discernable effect on their development in the thymus. These findings show that all leukocyte lineages exhibit flippase activity, and identify ATP11C as an aminophospholipid translocase in immune cells.

## Introduction

The plasma membrane in eukaryotes envelops cells and consists of a bilayer structure of glycerophospholipids, sphingolipids and cholesterol [1] with embedded proteins. One of the unique features of the plasma membrane is the asymmetric distribution of specific phospholipids between the two leaflets of the bilayer [2]. For instance, phosphatidylcholine (PC) and sphingomyelin are predominantly present on the exoplasmic leaflet, while phosphatidylserine (PS) and phosphatidylethanolamine (PE) are mainly confined to the cytoplasmic leaflet of the plasma membrane [3]. The establishment and dynamic maintenance of the non-random distribution of phospholipids is important for normal membrane functions, and has been implicated in many cellular processes including blood coagulation, vesicle formation and apoptosis [3].

Asymmetry of the plasma membrane is actively generated by two groups of ATP-dependent transporters embedded in the membrane. ATP-binding cassette (ABC)-type transporters (floppases) are responsible for active transport of specific lipids, such as PC, to the exoplasmic leaflet [4], while flipping of PS and PE into the inner leaflet of the plasma membrane is mediated by the P4-type ATPases (flippases) [5–8]. Although the biochemical activity of flippases has been known for a long time, their identification and characterization is still incomplete. In 1996, Tang et al. showed in yeast that a Drs2 null mutant is defective in ATP-dependent transport of NBD-labeled PS [9]. Subsequent studies in yeast revealed that Dnf1 and Dnf2, two other members of the P4-type ATPases, reside in the plasma membrane and mediate the transport of NBD-labeled PE, PS and PC from the outer to the inner leaflet of the plasma membrane [10]. Recently, Drs2 has also been shown to directly mediate the translocation of PS [11]. In eukaryotes, the Arabidopsis protein ALA1, which is homologous with Drs2 in yeast [12] was identified as a phospholipid flippase and in *Caenorhabditis elegans* germ cells the P4-type ATPase TAT-1 was shown to facilitate the inward transport of PS [13]. Mammalian flippases ATP8A1, ATP8A2, ATP8B1, ATP8B3 and ATP10A have been shown to be involved in the translocation of phospholipids between the two leaflets of the bilayer [14–18]. These findings collectively suggest that members of the P4-type ATPase family have the ability to translocate specific phospholipids from the exoplasmic leaflet to the cytoplasmic leaflet of biological membranes, thereby acting as a flippase.

A third group of transporters, known as scramblases, are believed to disrupt lipid asymmetry. In contrast to energy-dependent flippases and floppases, scramblases facilitate bidirectional movement of all types of phospholipids, but require activation often depending on elevation of the intracellular Ca^2+^ concentration or the induction of apoptosis [19].

Despite the importance of lipid transporters, their characterization and function, particularly in cells of the immune system, remains mostly unknown. We and others previously reported that *N*-ethyl-*N*-nitrosourea (ENU)-induced mutations in the murine *Atp11c* gene result in B cell deficiency due to a developmental arrest at the pro-B cell stage of B lymphopoiesis in the bone marrow [20, 21]. ATP11C has been subsequently reported in mice to play a critical role in erythrocyte longevity and morphology [22], as well as bile secretion [23]. Moreover, during apoptosis ATP11C undergoes limited proteolysis to facilitate exposure of PS [24]. Our initial measurements of PS internalization by different types of hematopoietic lineages revealed only relatively modest differences in flippase activity between control and ATP11C-deficient pro-B cells as well as double-negative (DN) and double-positive (DP) thymocytes [20]. However, with the use of a more sensitive PS analog, C_6_-NBD-PS, we recently showed that erythroblasts from mutant mice also exhibit severely reduced flippase activity compared to corresponding cells from control animals [22]. Using the C_6_-NBD-PS analog as well as fluorescently labeled PE and PC we examined in this study i) the ability of major leukocyte subsets to translocate specific phospholipids between the bilayer of the plasma membrane, and ii) whether the P4-type ATPase ATP11C is involved in this aminophospholipid translocation activity.

## Materials and Methods

### Mice

The mouse strain with an X-linked ENU-induced point mutation in *Atp11c* has been described previously [20]. This strain was maintained either by breeding heterozygous females with wild-type littermates or with wild-type C57BL/6 males, and ATP11C mutant and wild-type male mice were used in the experiments. Heterozygous females were also crossed with C57BL/6-SJL.Ptpc males in order to obtain mutant mice congenic for CD45.1. All experimental mice were housed in specified pathogen-free conditions at the Australian Phenomics Facility, and all animal procedures were approved by the Australian National University Animal Ethics and Experimentation Committee.

### Cell Preparation

The mice were sacrificed by cervical dislocation. Bone marrow, spleen and thymus were collected into tissue culture medium prepared as described previously [25]. Bone marrow cells were extracted by pressurized flow of buffer through dissected femurs and tibias. Single cell suspensions from spleen and thymus were prepared by passing the tissues through 70 μm nylon mesh filters (BD Biosciences). Red blood cells (RBC) in the spleen samples were removed by incubating splenocytes with RBC lysis buffer (150 mM NH_4_Cl, 10 mM KHCO_3_, 0.1 mM EDTA, pH 7.3). White blood cells were counted using the ViCELL cell counter (Beckham Coulter Inc.).

### Aminophospholipid Translocase (Flippase) Activity Assay

**Fig 1:**
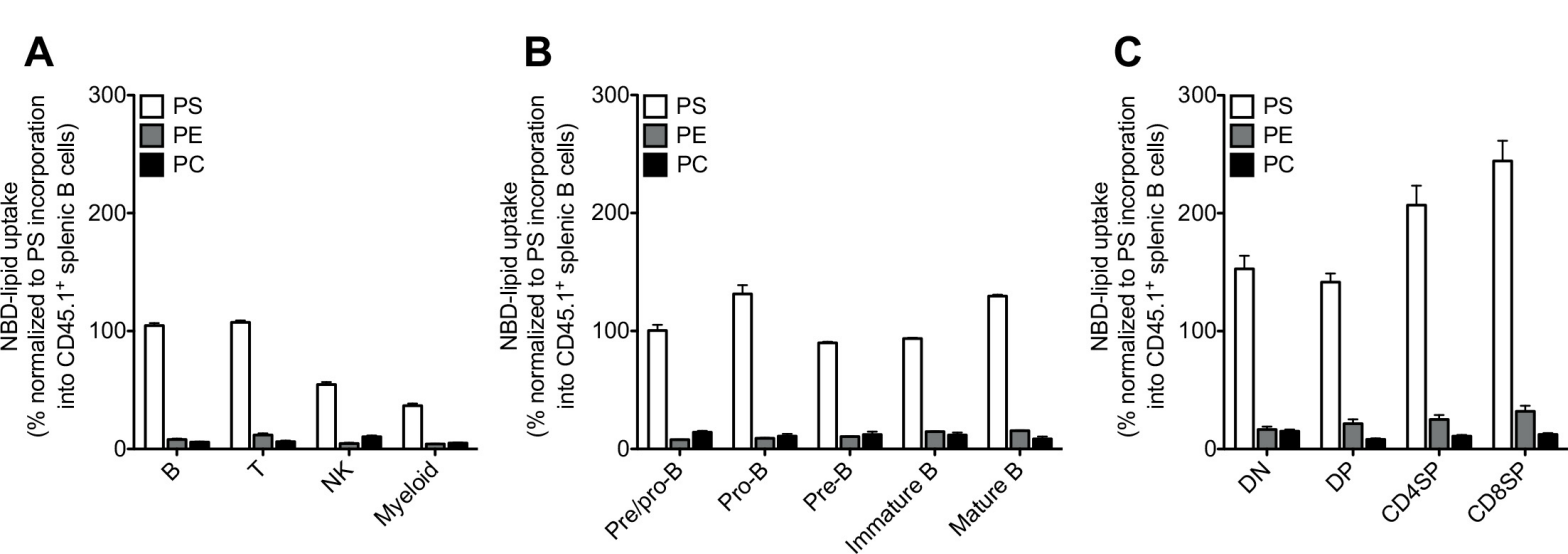
Leukocytes exhibit aminophospholipid translocation activity. Graphs represent mean ± S.E.M. of NBD-PS, NBD-PE or NBD-PC uptake into different immune cell subsets in the spleen (A), different B cell subsets in the bone marrow (B), and different T cell subsets in the thymus (C) of wild-type mice after 20 min of incubation. Data are presented as percentage of NBD-lipid uptake into the indicated subset relative to NBD-PS uptake into CD45.1-marked wild-type splenic B220^+^ cells stained and analyzed in the same tube (PS) or for an identical time in a separate tube (PE and PC). Cell populations were identified as follows. In the spleen: B cells (B220^+^), T cells (TCRβ^+^), NK cells (NK1.1^+^) and myeloid cells (CD11b^+^); in the bone marrow: Pre/pro-B cells (CD24^−^CD43^+^ in the B220^lo^IgM^−^ gate), pro-B cells (CD24^int^CD43^+^ in the B220^lo^IgM^−^ gate), pre-B cells (CD24^hi^CD43^−^ in the B220^lo^IgM^−^ gate), immature B cells (B220^lo^IgM^+^ gate) and mature B cells (B220^hi^IgM^+^ gate); and in the thymus: double negative (DN) cells (CD4^–^CD8^–^), double positive (DP) cells (CD4^+^CD8^+^), CD4 single positive (SP) cells (CD4^+^CD8^–^) and CD8SP cells (CD4^–^CD8^+^). DAPI^+^ dead cells were excluded from the analysis. Data are representative of two independent experiments with two to three mice in each. Graphs represent mean values ± S.E.M. from three biological replicates.

Flippase activity assay was performed with mutant and wild-type bone marrow, spleen and thymic cells using the following fluorescent lipid analogues: 1-palmitoyl-2-{6-[(7-nitro-2-1,3-benzoxadiazol-4-yl)amino]hexanoyl}-*sn*-glycero-3-phosphoserine (ammonium salt) (C_6_-NBD-PS), 1-palmitoyl-2-{6-[(7-nitro-2-1,3-benzoxadiazol-4-yl)amino]hexanoyl}-sn-glycero-3-phosphoethanolamine (C_6_-NBD-PE), 1-palmitoyl-2-{6-[(7-nitro-2-1,3-benzoxadiazol-4-yl)amino]hexanoyl}-sn-glycero-3-phosphocholine (C_6_-NBD-PC) (all from Avanti Polar Lipids, Inc.). Cells were washed with Hanks’ Balanced Salt Solution (HBBS, pH 6.0) containing 1 mM MgSO_4_ and 1.3 mM CaCl_2_. Cells were then incubated with 5 μM NBD-labeled lipids in 200 μl of the same solution for 20 minutes. To minimize unspecific uptake of phospholipids via endocytosis, all incubation steps were performed at 15°C [26]. To stop the assay (flipping of lipid analogs) and to remove unbound analogs from the cell surface, cells were placed on ice and incubated with HBBS (pH 7.4) containing 1 mM MgSO_4_, 1.3 mM CaCl_2_ and 1% fatty acid free bovine serum albumin (BSA) (Sigma-Aldrich) for 5 min. Cells were then pelleted by centrifugation and re-incubated with 1% fatty acid free BSA followed by centrifugation. Cells were then washed twice in HBBS (pH 7.4) containing 1 mM MgSO_4_ and 1.3 mM CaCl_2_, and transferred to a 96-well plate to be stained with fluorescently labeled antibodies as described below. The data presented in Fig 1 were calculated based on the following formula:

$$\text{NBD-lipid uptake}\;\left(\%\right)=(\text{geometric mean fluorescence intensity}\;\left(\text{MFI}\right)\;\text{of NBD-PS},\;\text{-PE or -PC uptake into CD}45.2^{+}\;\text{subsets}\;/\;\text{geometric MFI of NBD-PS uptake into CD}45.1^{+}\;\text{splenic B cells})\;\text{x}\;100.$$

### Surface Antibody Staining

Cells were incubated with an antibody cocktail containing an appropriate combination of the antibodies each diluted to its optimal concentration in flow cytometry buffer (phosphate buffered saline supplemented with 2% bovine serum and 0.1% sodium azide) and incubated for 30 min at 4°C in the dark. Samples were washed and resuspended in flow cytometry buffer and analyzed on a LSR II or LSR Fortessa flow cytometer (BD Biosciences).

For flow cytometric analysis the following antibodies were used: CD11b-APC-Cy7 (Clone M1/70, Biolegend), CD21/35-APC (Clone 7E9, Biolegend), CD21/35-Biotin (Clone 7E9, Biolegend), CD23-Pacific Blue (Clone B3B4, Biolegend), CD23-PE Cy7 (Clone B3B4, eBioscience), CD24-Biotin (Clone 91, Southern Biotech), CD4-PE Cy7 (Clone RM4-5, BD), CD43-APC (Clone 1B11, Biolegend), CD45.1-Alexa Fluor 700 (Clone A20, Biolegend), CD45.2-BUV737 (Clone 104, BD), CD45.2-Pacific Blue (Clone 104, Biolegend), CD45R/B220-APC Cy7 (Clone RA3-6B2, BD), CD8-APC (Clone 53–6.7, eBioscience), CD8-PE Cy7 (Clone 53–6.7, Biolegend), CD93-APC (Clone AA4.1, eBioscience), CD93-Biotin (Clone AA4.1, eBioscience), IgM-PE Cy7 (Clone II/41, eBioscience), NK1.1-APC (Clone PK136, BD), TCRβ-Biotin (Clone H57-597, BD), Streptavidin Brilliant Violet 605 (Biolegend), Streptavidin-Qdot 605 (Invitrogen), 4´,6-diamidino-2-phenylindole, dilactate (DAPI) (Thermo Fisher Scientific) and 7-Aminoactinomycin D (7-AAD) (Thermo Fisher Scientific).

### Annexin-V Staining

Cell suspensions were prepared, counted and equal numbers of cells were allowed to rest for 2h in tissue culture medium at 37°C and 5% CO_2_. For analysis of Annexin-V staining without resting, aliquots of freshly isolated cells were dispensed into the 96-well round-bottom plates after preparation of single cell suspensions. Cells were then stained with the appropriate antibodies as detailed above and were washed once with flow cytometry buffer and once with Annexin-V binding buffer (Biolegend). Pacific Blue conjugated Annexin-V (Biolegend) staining was performed in Annexin-V binding buffer at room temperature for 15 min and washed and resuspended in Annexin-V binding buffer and kept at 4°C until analysis.

### Statistical Analysis

Multiple experimental groups were compared using One-Way Analysis of Variance (ANOVA), followed by pair-wise comparison with a Bonferroni post-test, and differences were taken to be significant when *P* < 0.05. All statistical analyses were performed using GraphPad Prism Software.

## Results

### Differential flippase activity by various hematopoietic lineages

We first wanted to test whether immune cell subsets translocate specific aminophospholipids between the two leaflets of the plasma membrane and compare the flippase activity between major subsets in different tissues. To do so, congenically-marked CD45.2^+^ cells from the spleen, bone marrow and thymus of wild-type animals were mixed with congenically-marked CD45.1^+^ wild-type spleen cells and incubated with lipid analogs, NBD-PS, NBD-PE or NBD-PC for 20 min, followed by antibody staining and analysis by flow cytometry. To facilitate the comparison between different tissues and different lipid analogs, the uptake of lipids in different subsets was then normalized to PS incorporation into CD45.1^+^ splenic B cells. As shown in Fig 1A, we observed that all major immune cell subsets display aminophospholipid flippase activity with PS being the most translocated lipid derivative compared to PE and PC (Fig 1A). Interestingly, measurement of PS internalization in different subsets in the spleen revealed that B and T cells have the highest PS uptake compared to other cells with CD11b^+^ myeloid cells showing the lowest flippase activity (Fig 1A). Consistent with the confinement of PE to the cytoplasmic leaflet of the plasma membrane, the internalization of PE was found to be slightly higher than that of PC in B and T cells in the spleen (Fig 1A).

We then compared the uptake of NBD-labeled PS, PE and PC in different B and T cell subsets during their development in the bone marrow and thymus, respectively. Analysis of B cell subsets in the bone marrow showed that pro-B cells have the highest level of PS internalization compared to other subsets such as pre/pro-B, pre-B, immature B and mature B cells (Fig 1B). By contrast, the uptake of PE and PC did not significantly differ in B cell subpopulations in the bone marrow (Fig 1B). Similarly, T cell subsets in the thymus had a variable requirement for PS translocation as the highest level of PS internalization was observed in CD8 single-positive (SP) and CD4SP cells compared to DN and DP subsets (Fig 1C). Interestingly, the uptake of PS and PE in thymic T cell subsets was the highest compared with other major immune subsets in the spleen and bone marrow (Fig 1A–1C).

Collectively, these results demonstrate that all major immune cell subsets exhibit aminophospholipid translocation activity especially for PS and that this activity varies in different subpopulations.

### ATP11C acts as a flippase in immune cell subsets

**Fig 2:**
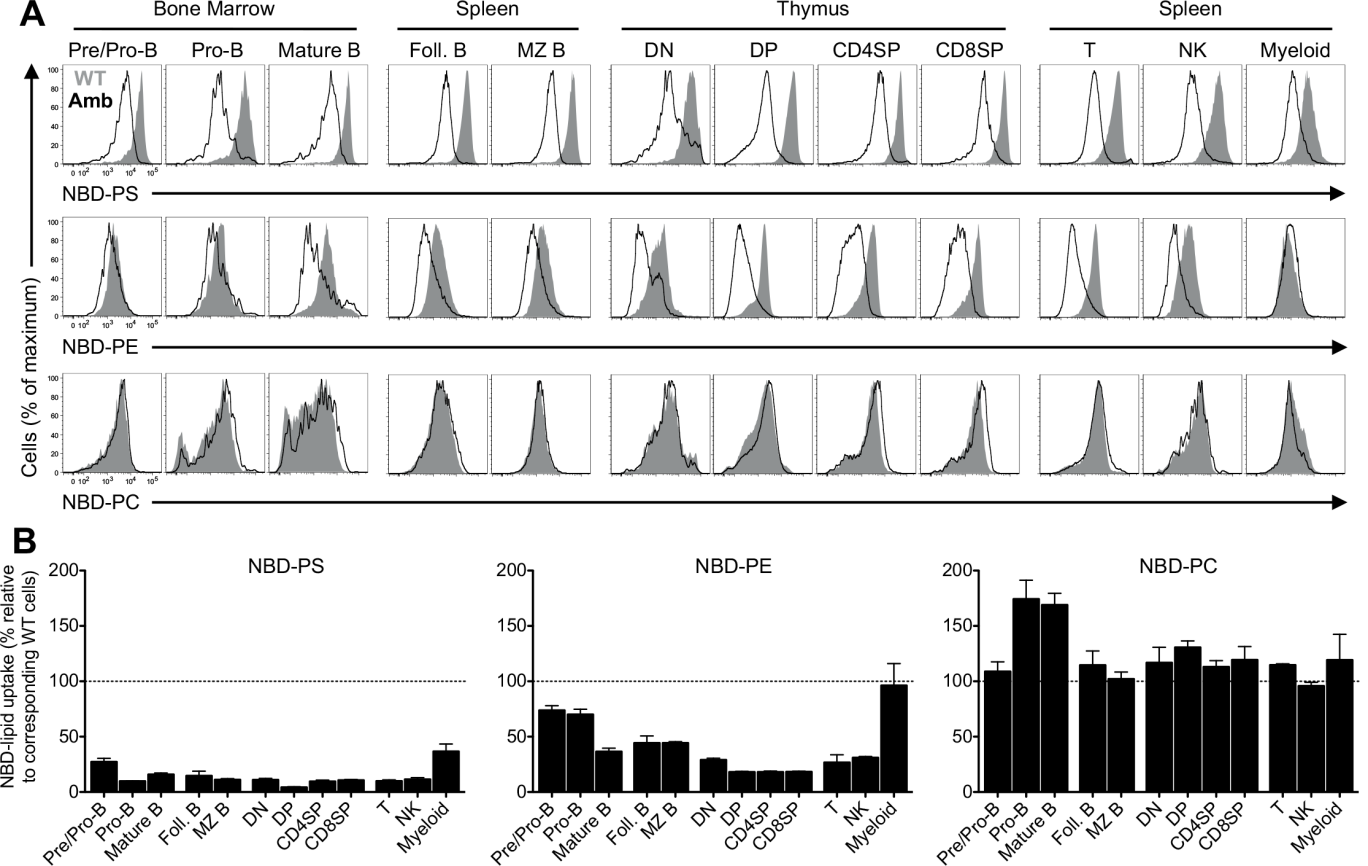
Immune cell subsets from ATP11C-deficient mice display a defective PS and PE flippase activity. (A) Representative overlay histogram of NBD-PS, NBD-PE and NBD-PC fluorescence profiles after 20 min incubation in pre/pro-B, pro-B and mature B cells in the bone marrow; follicular (Foll.) and marginal zone (MZ) B cells in the spleen; DN, DP, CD4SP and CD8SP cells in the thymus; and T, NK and myeloid cells in the spleen of *Atp11c*^*amb/0*^ (Amb, black lines) animals, compared to the corresponding CD45.1- or CD45.2-marked wild-type cells in the same tube (WT, shaded grey). (B) Graphs represent mean ± S.E.M. of the percentage of NBD-lipid uptake relative to the corresponding wild-type cells in the same tube. Follicular B cells (CD21^int^CD23^+^ in the B220^+^CD93^−^ mature B cell gate) and MZ B cells (CD21^hi^CD23^−^ in the B220^+^CD93^−^ mature B cell gate) in the spleen, other cell types as described in Fig 1. DAPI^+^ dead cells were excluded from the analysis. Data are representative of at least four independent experiments with one to three mice in each.

Because the P4-type ATPase ATP11C has been shown to translocate PS in developing B cells in the bone marrow [20] and developing erythroblasts in the bone marrow and spleen [22], we decided to test if the observed flippase activity in all leukocyte subsets is ATP11C-dependent. To test this, *Atp11c*^*amb/0*^ cells from either CD45.1 or CD45.2 animals were mixed at a 1:1 ratio with cells from wild-type CD45.2 or CD45.1 animals respectively and incubated with lipid analogs as detailed above. Given that loss of ATP11C results in a B cell deficiency syndrome caused by a developmental block in early B cell development [20, 21], we first focused on B cell subsets in the bone marrow. All B cell subsets including pre/pro-B, pro-B and mature B cells from the bone marrow of *Atp11c*^*amb/0*^ animals showed a significantly reduced level of PS internalization compared to those from *Atp11c*^*+/0*^ animals (Fig 2A and 2B). The lower internalization of PS in ATP11C-deficient cells was observed after a short incubation period (1 min) and persisted up to 60 min (data not shown). Likewise, mature follicular and marginal zone B cells in the spleen of *Atp11c*^*amb/0*^ animals had reduced levels of PS uptake (Fig 2A and 2B).

To determine whether the defective flippase activity is specific for B cell subsets, we tested the uptake of PS into different leukocyte subsets including developing T cells in the thymus, mature T cells, NK cells and myeloid cells in the spleen. Analysis of different T cell subsets in the thymus demonstrated that DN, DP, CD4SP and CD8SP thymocytes from ATP11C-deficient animals showed a significantly reduced PS internalization compared to the corresponding thymocytes in wild-type mice (Fig 2A and 2B). A similar reduction of flippase activity was also observed in T cells, NK cells and myeloid cells from the spleen of *Atp11c*^*amb/0*^ animals (Fig 2A and 2B).

The P4-type ATPases have been suggested to transport mainly PS, but also to a lesser extent PE [5–7]. Consistent with this notion, all *Atp11c*^*amb/0*^ immune cell subsets were also deficient in translocating PE to the cytoplasmic leaflet of the plasma membrane with the possible exception of CD11b^+^ myeloid cells (Fig 2A and 2B). The loss of flippase activity varied from 18% to 73% in different subsets with a more severe reduction in T cells in the thymus and spleen of mutant mice (Fig 2A and 2B). The incorporation of PE into ATP11C-deficient CD11b^+^ myeloid cells seemed to be comparable to control cells (Fig 2A and 2B), but this could simply be due to a relatively low PE internalization in this subset (Fig 1A). By contrast, the internalization of PC in immune cell subsets from ATP11C-deficient mice was largely comparable to their counterparts from control animals (Fig 2A and 2B).

Taken together, these data show that ATP11C is a flippase that selectively mediates the inward transport of PS and PE across the plasma membrane of immune cell subsets, and that cells from ATP11C-deficient animals are defective in the internalization of PS and PE.

### Defective flippase activity causes accumulation of PS in the outer plasma membrane leaflet of viable 7-AAD^−^ pro-B and T cells from ATP11C-deficient animals

**Fig 3:**
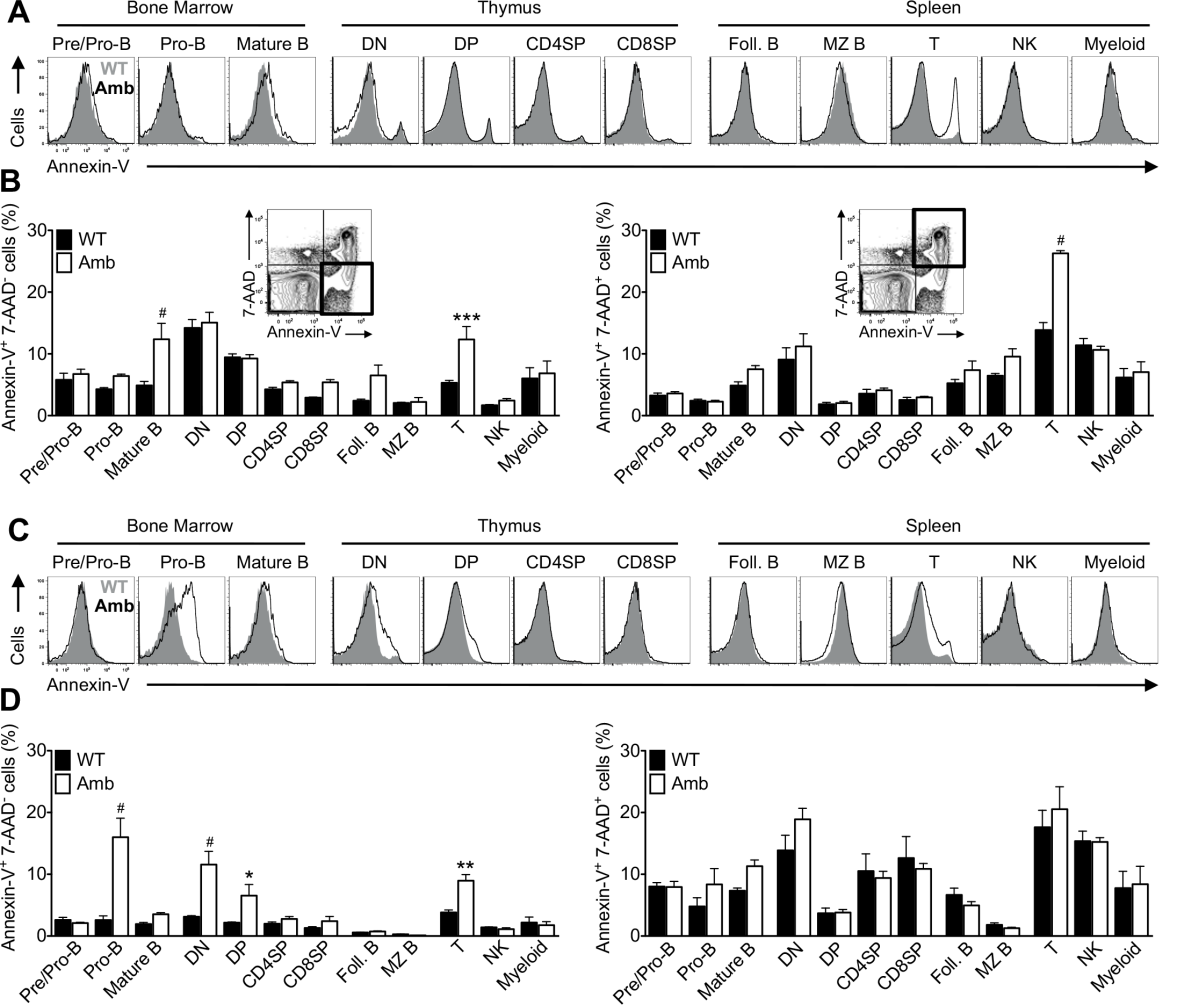
Increased PS exposure on viable 7-AAD^−^ pro-B and T cells from ATP11C-deficient animals. (A) Equal numbers of cells from bone marrow, thymus and spleen of *Atp11c*^*+/0*^ and *Atp11c*^*amb/0*^ animals were allowed to recover in tissue culture medium for 2h followed by testing for Annexin-V binding. Representative overlay histogram of Annexin-V staining in pre/pro-B, pro-B and mature B cells in the bone marrow; DN, DP, CD4SP and CD8SP cells in the thymus; and Foll. B, MZ B, T, NK and myeloid cells in the spleen of *Atp11c*^*+/0*^ (WT, gray area) and *Atp11c*^*amb/0*^ (Amb, black line) animals. Cells were pre-gated on 7-AAD^−^ cells. Data are representative of three independent experiments with two to three mice per genotype in each. (B) Graphs represent mean ± S.E.M. of the percentage of Annexin-V^+^7-AAD^−^and Annexin-V^+^7-AAD^+^ cells in different subsets, gated as in micro flow cytometric panels. Data are pooled from two independent experiments with two to three mice per genotype in each. (C) Equal numbers of cells from bone marrow, thymus and spleen of *Atp11c*^*+/0*^ and *Atp11c*^*amb/0*^ animals were directly tested for Annexin-V binding without being cultured. Representative overlay histogram of Annexin-V staining in pre/pro-B, pro-B and mature B cells in the bone marrow; DN, DP, CD4SP and CD8SP cells in the thymus; and Foll. B, MZ B, T, NK and myeloid cells in the spleen of *Atp11c*^*+/0*^ (WT, gray area) and *Atp11c*^*amb/0*^ (Amb, black line) animals. Cells were pre-gated on 7-AAD^−^ cells. Data are representative of four independent experiments with two to five mice per genotype in each. (D) Graphs represent mean ± S.E.M. of the percentage of Annexin-V^+^7-AAD^−^and Annexin-V^+^7-AAD^+^ cells in different subsets, gated as in (B). Data are pooled from two independent experiments with two to three mice per genotype in each. Statistics were calculated using One-Way ANOVA, followed by pair-wise comparison with a Bonferroni post-test. * *P* < 0.05, ** *P* < 0.01, *** *P* < 0.001, ^#^ *P* < 0.0001.

Exposure of PS on the surface of cells is critical for the recognition and clearance of apoptotic cells by macrophages [27]. The impaired flippase activity in immune cell subsets from ATP11C-deficient animals raises the possibility that the ATP11C mutation leads to an accumulation of PS in the exoplasmic plasma membrane leaflet of cells. To test this hypothesis, freshly isolated cells from *Atp11c*^*amb/0*^ and *Atp11c*^*+/0*^ control animals were allowed to recover in tissue culture medium for 2h before staining with Annexin-V, which binds to PS [28], followed by analysis on a flow cytometer. Of all analyzed viable 7-AAD^−^ cell populations in the bone marrow, thymus and spleen only mature B cells in the bone marrow showed a small shift in Annexin-V binding (Fig 3A), suggesting that the ATP11C deficiency does not result in a general steady-state increase in PS exposure on the surface of cells. We also found an increase in the percentage of Annexin-V^+^7-AAD^−^ early apoptotic and Annexin-V^+^7-AAD^+^ apoptotic T cells in the spleen (Fig 3A and 3B). These results are consistent with the recent findings that ATP11C-deficient cells do not generally have increased PS exposure on their surface [24], suggesting the presence of additional flippases in the membrane of immune cells. In agreement with this notion, PS exposure is increased in cells deficient of CDC50A, the trafficking subunit for a variety of P4-type ATPases [24].

Surprisingly, when freshly isolated cells were directly tested for Annexin-V fluorescence without recovery in tissue culture medium, a fraction of viable 7-AAD^−^ pro-B cells in the bone marrow, DN and DP thymic T cell subsets as well as mature T cells in the spleen from mutant animals had elevated Annexin-V binding (Fig 3C and 3D).

**Fig 4:**
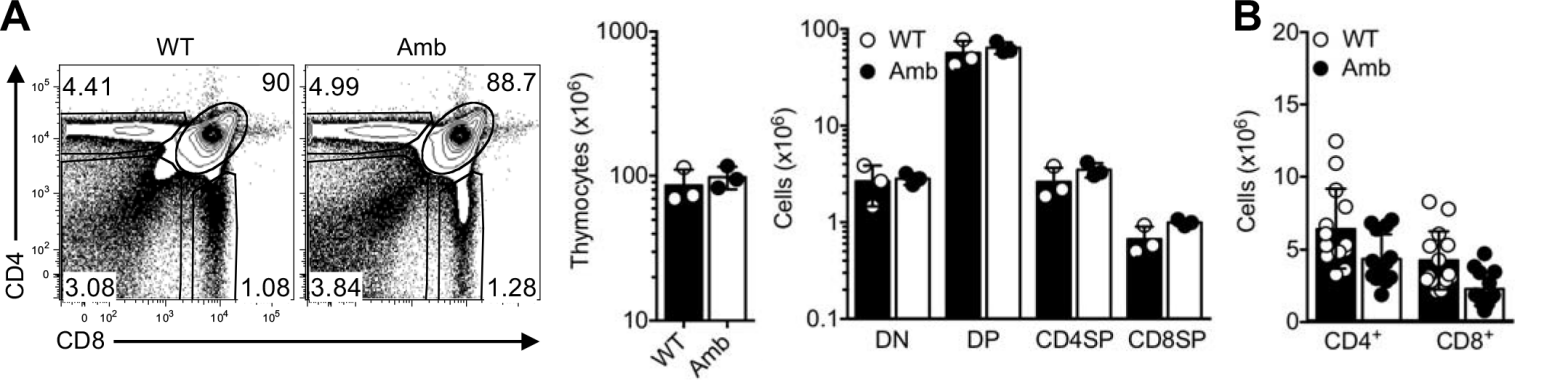
Normal T cell development in the thymus of ATP11C-deficient mice. (A) Representative flow cytometric analysis of T cells from the thymus of *Atp11c*^*+/0*^ (WT) and *Atp11c*^*amb/0*^ (Amb) animals. Graphs show the absolute number of total thymocytes (left) and different T cell subsets (right) in the thymus of *Atp11c*^*+/0*^ (WT) and *Atp11c*^*amb/0*^ (Amb) animals. (B) Graph shows the absolute number of CD4 and CD8 T cells in the spleen of *Atp11c*^*+/0*^ (WT) and *Atp11c*^*amb/0*^ (Amb) animals. Each symbol represents an individual mouse and bar graphs represent mean ± S.E.M. Data are representative of three independent experiments with three to four mice per genotype in each (A) or are pooled from four independent experiments with three to five mice per genotype in each (B).

The increased Annexin-V binding on freshly isolated developing and mature T cells and the increased percentage of apoptotic T cells in the spleen raises the possibility that ATP11C could have a role in T cell development or survival. However, T cell development in the thymus was normal (Fig 4A) and, consistent with previous reports, the number of T cells in the periphery of mutant mice remained largely unaffected (Fig 4B) [20]. The observed small reduction in splenic T cells in Fig 4B can most likely be attributed to the reduced number of splenic B cells in ATP11C-deficient mice. This is also in agreement with the finding of normal percentages of ATP11C-deficient T cells in the spleen of mixed bone marrow chimeras [20, 21]. One explanation for normal T cell development in the thymus and their persistence in the periphery despite increased Annexin-V staining on T cells could be that this is a temporary observation after isolation and that normal asymmetric distribution of PS can be maintained or re-established *in vivo* through other flippases.

Collectively, the results presented here demonstrate that a null-mutation in the gene encoding ATP11C results in markedly reduced flippase activity in immune cell subsets, and shows that ATP11C is a flippase that is crucial for the transport of lipids from the outer leaflet of the plasma membrane to the inner leaflet. The data also show phospholipid specificity of ATP11C as cells lacking ATP11C failed to flip PS and to a lesser extend PE, but not PC, from the exoplasmic to the cytoplasmic leaflet of the bilayer cells.

## Discussion

The results presented in this study revealed that immune cell subsets exhibit variable phospholipid internalization profiles, especially towards PS. The simplest explanation for the variable internalization profiles by different subsets is the differential expression of flippases in immune cell subsets [29]. Why different cell types express different levels of the flippases is unclear but one possibility is that they may need higher levels of flippase activity at specific stages of their development to maintain the normal asymmetric distribution of the different phospholipids.

Our results show that the P4-type ATPase ATP11C translocates specific aminophospholipids between the two leaflets of the plasma membrane in immune cell subsets. Loss of ATP11C resulted in a defective flippase activity toward PS and PE. In contrast to our initial report [20], where only pro-B, DN and DP cells from *Atp11c*^*amb/0*^ mice were found to have a reduced level of PS internalization compared to those from control animals we describe here a more global defect in flippase activity in all major immune cell subsets. Our results demonstrate that flippase activity is regulated during immune cell development and differs between subsets. However, in each subset more than 75% PS flippase activity appears to be mediated by ATP11C. In DP T cells in the thymus, ATP11C mediated about 95% of PS flipping. The difference to our previous observation is most likely caused by the use of a more sensitive analogue (C_6_-NBD-PS) that differs from the original C_12_-NBD-PS ligand by the length of the *sn-2* chain to which the NBD fluorescence marker is attached. The shorter *sn-2* chain increases flippase-mediated transbilayer movement and allows back-extraction using fatty acid free BSA [30]

ATP11C-deficient animals have a specific defect in early B cell development in the bone marrow [20, 21]. One explanation for the pro-B cell specific defect in development despite the widespread loss of flippase activity in immune cell subsets could be that these cells rely more on flippase activity compared to other cells because of an intrinsically increased PS externalization. Consistent with a delicate balance between PS exposure and internalization, viable pro- and pre-B cells have been shown to express slightly increased levels of PS on their surface [31, 32]. This is not caused by a loss of flippase activity at the pro-B cell stage because our data show that pro-B cells have the highest PS internalization level of all developing B cell subsets (Fig 1). However, the variable internalization in different B cell subsets does not correlate with the largely similar gene expression of *Atp11c* in different B cell subpopulations in the bone marrow [29].

The increased exposure of PS on freshly isolated cells was particularly pronounced on pro-B cells lacking ATP11C. As discussed above, this could reflect a high reliance of pro-B cells on flippase activity for maintenance of phospholipid asymmetry and may result in an increased vulnerability compared to other B cell subsets during lymphopoiesis in the bone marrow. Since exposure of PS on the surface of cells is critical for the recognition and clearance of apoptotic cells by macrophages [27], an alternative explanation for the stage specific defect in B cell lymphopoiesis could be that the impaired flippase activity causes a temporary increased PS concentration on the cell surface, which in turn could result in rapid PS-mediated phagocytosis of B cells in ATP11C-deficient mice.

Our finding of elevated Annexin-V on freshly isolated live pro-B cells suggests that during the preparation of cell suspensions, cells may undergo some mechanical stress that leads to increased exposure of PS on the outer leaflet [33], possibly through the activation of scramblases in viable 7-AAD^−^ pro-B cells in the bone marrow, as well as on DN and DP T cell subsets in the thymus. However, when cells were rested for 2 hours they showed no increased Annexin-V staining, presumably because there were able to re-establish PS asymmetry through the activity of other flippases. The apparent contradiction between severely reduced PS internalization in the absence of ATP11C (Fig 2) and largely normal exposure of PS in most lymphocyte subsets (Fig 3) can possibly be explained by the difference between measuring exposure of endogenous PS under steady state conditions and measuring the uptake of exogenous PS.

In conclusion, the results presented in this study demonstrate for the first time that a mutation in the gene encoding ATP11C resulted in the diminished flippase activity in all immune cell subsets, and suggest that ATP11C is a flippase that selectively transports PS and PE from the outer leaflet of the plasma membrane to the inner leaflet. These findings extend our initial results of defective flippase activity in ATP11C-deficient pro-B cells [20] and erythroblasts [22], and are in line with the subsequent findings reported recently in cell lines [24, 34].
